# The legacy of a gentleman scientist: Pierre Hainaut

**DOI:** 10.1038/s41418-025-01586-5

**Published:** 2025-10-03

**Authors:** Christophe Arnoult, Laura D. Attardi, Kerem Batsheva, Giovanni Blandino, Kathleen H. Burns, Giannino Del Sal, David G. Kirsch, David P. Lane, Arnold J. Levine, Guillermina Lozano, David Malkin, Gerry Melino, Moshe Oren, Carol Prives, Daniel Schramek

**Affiliations:** 1 Institute for Advanced Biosciences (IAB), INSERM 1209, La Tronche, France; 2 Stanford University School of Medicine, Stanford, CA USA; 3 The Hebrew University, Jerusalem, Israel; 4 Regina Elena National Cancer Institute, IRCCS, Rome, Italy; 5 Dana-Farber Cancer Institute, Boston, MA USA; 6 International Centre for Genetic Engineering and Biotechnology, Trieste, Italy; 7 Princess Margaret Cancer Centre, Toronto, ON Canada; 8 Karolinska Institutet, Biomedicum, Stockholm, Sweden; 9 Institute for Advanced Study, Princeton, NJ USA; 10 The University of Texas MD Anderson Cancer Center, Houston, TX USA; 11 Hospital for Sick Children, Toronto, ON Canada; 12 Tor Vergata University of Rome, Rome, Italy; 13 Weizmann Institute of Science, Rehovot, Israel; 14 Columbia University, New York, NY USA; 15 Lunenfeld-Tanenbaum Research Institute, Toronto, ON Canada

**Keywords:** Tumour-suppressor proteins, Cancer genetics, Cancer genetics

**Figure:**
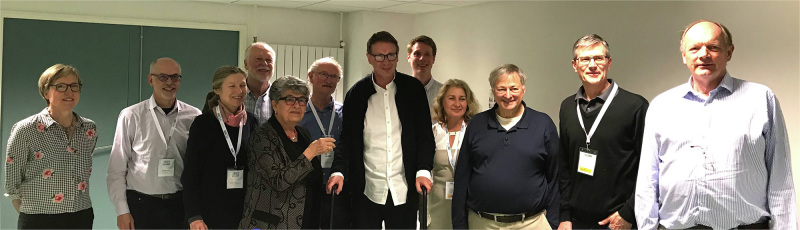
Pierre with his friends in 2019 in Lyon.

*“To live is the rarest thing in the world*.

*Most people exist, that is all.”*

— Oscar Wilde

It is with immense sadness that we share the heartbreaking news of the passing of Professor Pierre Hainaut, who died while hiking in the mountains of Italy on July 31, 2025, at the age of 67.

Today we remember one of the most brilliant minds of his generation, a pillar of cancer research, but above all, a human being of rare nobility; definitively, Pierre did not simply “exist”, he had an intense life that has left a generous legacy to all of us and inspiration for generations to come. Pierre was ‘*one of a kind’* in the deepest and broadest sense of these words. His passion and enthusiasm were overwhelming, making a huge impact in the scientific community as well as at an international level. He was also a wonderful human being with a rich culture, not only in science but, indeed, also in culture, history and the humanities. With his generous spirit, he was a good friend to many. Pierre was a visionary, and shared his vision with leading scientists and clinicians, friends and students with passion and deep knowledge on an amazing breadth of subjects. His deep analytical insight and ability to synthesize ideas commanded both admiration and respect. Indeed, Pierre was widely respected for his scientific excellence, human qualities, and strategic vision. A man of great intellect and remarkable humility, he combined a broad perspective with profound ability to listen. These are quality of a great mentor. His vast clinical knowledge, his wit, his friendliness, his tenacity, will be greatly missed by students, colleagues, friends and collaborators in the scientific community. To those who had the privilege of working alongside him, Pierre will be remembered as a deeply kind, passionate, and attentive colleague. His humility and tireless commitment to research and to his colleagues left an enduring mark on everybody who knew him.

Originally from Liège, Belgium, Pierre was an exceptional biologist, internationally recognized for his work on cancer, and in particular on the p53 protein. From 1999 to 2012, he served as Head of the Section of Mechanisms of Carcinogenesis at the International Agency for Research on Cancer (IARC), leading major efforts in cancer prevention particularly in low- and middle-income countries. He subsequently served as Director of the Institute for Advanced Biosciences for nine years, until January 2025, when he chose to step down to dedicate himself to leading the Cancéropôle Lyon Auvergne-Rhône-Alpes. His dedication to the Institute for Advanced Biosciences was total, and his leadership leaves a lasting legacy.

Pierre began his research career in studies of the p53 DNA binding domain, showing that zinc was required to fold and coordinate the structure and function of this domain. This was a prelude to understanding the most common *TP53* mutation, R175H. Over the years Pierre established himself as “the p53 epidemiologist”, helping to assemble databases of *TP53* mutations, both spontaneous and inherited. He helped to classify the types of tumors common to Li- Fraumeni families and most importantly to carry out a systematic characterization of the entire spectrum of p53 mutant alleles, selecting them into categories ranging from highly pathogenic to hypomorphs. For each class he listed age of onset and frequency of specific tumor types, and showed that there was an age dependent and sex dependent tumor profile over a lifespan. More recently, he showed that some mutants could give rise to HLA-presented neoantigens, a fact that may have major impact on cancer treatment and improved response to immunotherapy. Altogether, Pierre’s depth of understanding and insights for future research were extraordinary and the impact of his discoveries will only increase as the first drugs able to “rescue” p53 mutants reach patients

Though he came from an urban background, Pierre had a profound love for nature and the mountains. He drew strength from hiking, cycling, and the silence of high altitudes. After suffering a severe cycling accident during a descent from Alpe d’Huez, he demonstrated extraordinary determination and raw courage in his recovery, ultimately returning to his leadership role and continuing to champion the scientific projects closest to his heart.

Pierre leaves behind a profound legacy, both scientific and human, that we will carry forward. The scientific community pays tribute to him and joins his family in mourning this tremendous loss. He was a dear friend and an inspiration for his generation: not only for of his professional achievements, but equally for his courage, his strong personality, his passion for science and for humanity, and for his infectious unbounded enthusiasm.

We will miss him sorely. Our thoughts and deepest sympathies are with his close beloved family, and his greatest love of all, his wife Françoise, his children Hadrien and Marie, and his grandchildren Léontine et Adèle at this tragic time.

